# Substandard antibiotics and their clinical outcomes among hospitalized patients in southern Malawi: a pilot study

**DOI:** 10.3389/fphar.2025.1535501

**Published:** 2025-03-18

**Authors:** Francis Kachidza Chiumia, Adamson Sinjani Muula, Frider Chimimba, Happy Magwaza Nyirongo, Elizabeth Kampira, Felix Khuluza

**Affiliations:** ^1^ Department of Pharmacy, Kamuzu University of Health Sciences, Blantyre, Malawi; ^2^ Department of Community and Environmental Health, Kamuzu University of Health Sciences, Blantyre, Malawi; ^3^ Department of Medical Laboratory Sciences, Kamuzu University of Health Sciences, Blantyre, Malawi

**Keywords:** substandard medicines, parenteral antibiotic medicines, clinical outcomes, adverse drug reactions, patient recovery

## Abstract

**Background:**

The burden of substandard antibiotics is high in low-middle income countries including Malawi. These poor-quality antibiotics may cause deleterious effects on patients and promote drug resistance. We assessed the quality of antibiotics and the associated clinical outcomes among hospitalized patients in southern Malawi.

**Methods:**

A cross-sectional study involving review of retrospective records was conducted among hospitalized adult patients at Zomba central, Machinga and Nsanje district hospitals in October 2022 and January 2024. Trained pharmacy personnel recorded the parenteral antibiotics that were issued to the medical wards. We used these records for matching and sampling of the administered medicine batches to the patient files. In total, we reviewed 224 patient management files for eligible patients, aiming to assess the patient recovery and the occurrence of adverse drug reactions (ADRs) using a global trigger tool. We collected nine medicine samples of ceftriaxone and benzylpenicillin which were administered to these patients and subjected them to tests for the content of active pharmaceutical ingredients using methods adapted from the United States Pharmacopeia. For each sample, we collected at least ten dosage units and used Agilent^®^ 1120 High Performance Liquid Chromatography for quality analysis.

**Results:**

Of the 224 reviewed files, ADRs occurred in 18.3% % (n = 41) of patients while 12.05% (n = 27) did not recover from their illness. One benzylpenicillin sample was found out of specifications with only 61.8% of declared amount of active ingredients. Among patients who received benzylpenicillin with optimal API content, 15.8% experienced ADRs while 10.5% failed to recover from illness. For patients who received benzylpenicillin containing lower than required amount of API, only 7.1% experienced an ADR while 14.3% failed to recover from illness. These differences were, however, not statistically significant. Patient outcomes were significantly associated with the patient’s age and Charlson comorbidity index (CCI), p < 0.05.

**Conclusion:**

The present findings did not reveal statistically significant differences in patient outcomes based on the assessed medicine quality. Therefore, we recommend a larger prospective study to further validate these results and encourage stakeholders to be more vigilant on the quality of antibiotic medicines, as this is a crucial measure for improving clinical outcomes and preventing antibiotic resistance in Malawi.

## Background

Substandard medicines pose a major risks to global public health (Ozawa et al., 2022a). The prevalence of substandard medicines varies by region and range from as low as <5% to as high as >50% (Ozawa et al., 2018) depending on medicine class, with studies reporting a disproportionally higher prevalence of substandard medicines in low-middle income countries (LMICs). Studies have reported an average prevalence of about 40% for substandard antibiotic medicines in Africa (Tegegne et al., 2024; Asrade Mekonnen et al., 2024) while for Asia, America and Europe, the reported prevalences of substandard antibiotics are lower and around 15%, 12% and 7% respectively (Zabala et al., 2022). Thus, the studies done in Africa, have consistently indicated that antibiotics face the highest burden of substandard medicines as compared to other medicine classes (Ozawa et al., 2022b; McManus and Naughton, 2020; Almuzaini et al., 2013; Akpobolokemi et al., 2022). On the other hand, antibiotic consumption is increasingly high in LMICs and estimated to reach up to 168 billion defined daily doses (DDDs) by 2030; a 200% increase from 42 billion DDDs in 2015 (Klein et al., 2018). This is a health concern as antibiotic medicines overuse increases the emergence of antibiotic resistance and occurrence of adverse drug reactions (ADRs). Antibiotic resistance is associated with the death of over 5 million people globally every year (Murray et al., 2022), while antibiotic-related ADRs contribute to more than 50% of individual case safety reports (Mudenda et al., 2016; Oleim et al., 2019).

Substandard medicines may contain incorrect amount of the declared content of active pharmaceutical ingredients (API), fail to release the API or contain unacceptable amount contaminants or impurities (Johnston and Holt, 2014). This may among other effects lead to sub-therapeutic exposure of antibiotics to bacteria and therefore further increase the risk of antibiotic resistance (Gulumbe and Adesola, 2023). Furthermore, substandard medicines are also associated with a higher risk of adverse drug events which eventually affect patient’s quality of life and adherence to treatment (Johnston and Holt, 2014). The substandard antibiotics increases the risk of death among patients by almost four-folds (Ozawa et al., 2022a). In 2017, the World Health Organization (WHO, 2024), estimated that, on the assumption of 10% prevalence of poor quality antibiotic medicines, about 72,000 deaths occurred due to lower activity of active ingredients and over 169,000 deaths occurred due to no drug activity among children presenting with pneumonia (World-Health-OrganizationWHO, 2017).

Previous studies conducted in Malawi have consistently shown a high prevalence of substandard antibiotic medicines ranging from 13%–45.5%, between 2014 and 2022. These studies mostly focused on oral antibiotics such as amoxicillin, ciprofloxacin, cefuroxime, co-trimoxazole, flucloxacillin and azithromycin (Khuluza et al., 2017; Khuluza, 2014; Chiumia et al., 2022). The burden of substandard medicines may still be under-estimated in Malawi as some of the samples in the reported studies were tested using less sensitive screening techniques such as thin layer chromatography (TLC) rather than pharmacopeial assay methods such as HPLC and ultraviolet/visible spectrometry (Pan and Ba-Thein, 2018).

Studies have also suggested that linkage of clinical outcomes to medicine batches using pharmacovigilance databases (for individual case safety reports) can offer an effective method for detection of possible poor quality medicines (Pozsgai et al., 2022; Juhlin et al., 2015). This study was therefore conducted to assess antibiotic ADRs and associate them with medicine quality to provide insights of safety signals which would be used as triggers for detection of possible substandard antibiotic medicines. With limited documented evidence on the clinical implications of poor-quality medicines among hospitalized patients, this study aimed to provide insights into the extent to which clinical outcomes are affected by substandard medicines.

## Methods

### Study design and setting

A cross-sectional study involving review of retrospective case management files for hospitalized patients who were prescribed antibiotic medicines was done in Zomba, Machinga and Nsanje districts in Malawi in October 2022 and January 2024. Malawi is one of the low-income countries located in Central Africa, bordering with Tanzania, Zambia and Mozambique. Among the serious health challenges affecting the country are high prevalence of infectious diseases and a critical shortage of trained healthcare professionals (World Health Organization, 2023a) (Muula, 2023). In 2020, the pharmaceutical personnel, physicians and nurses densities were 0.078, 0.494 and 7 per 10,000 population respectively (World Health Organization, 2023b). The study districts were randomly selected in Microsoft Excel 2019 version among 13 districts in the southern region of Malawi as described in another publication by our team which was addressing other objectives (Chiumia et al., 2022).

### Study population and sampling

We retrospectively reviewed case management files for patients who were administered with parenteral antibiotics and whose batch was traceable for sampling. The sample size was calculated using a single population proportion formula: n = p (1-p) *Z^2^/d^2^ (Sendekie et al., 2023). Using an estimated prevalence of 19% for antibiotic associated adverse events as reported by a study done in Uganda (Kiguba et al., 2017), a margin of error or 0.05 and Z value of 1.96, the sample size was 236 patient files. However, due to limited availability of eligible patient files, we included 224 patients in our analysis representing 95% of the required sample size. Trained pharmacy personnel kept records of the dates on which parenteral antibiotic medicines were issued to the adult medical wards. We used these records for matching and sampling of the medicine batches as well as selection of patient files for inclusion in the study. We sampled the medicine batch based on availability on the shelf and traceability of the patient files who were administered with the same medicine batch. The medicines in Malawian public hospitals are supplied monthly through a centralized system. However, due to short supply, some medicines may be out of stock for some days before the next consignment arrives (Chiumia et al., 2023; Khuluza and Heide, 2017; Chiumia et al., 2024). All re-admissions were excluded from the study. To avoid confusion, we also excluded patients on multiple antibiotic medicines, if one of the antibiotics was not traceable for sampling and testing. Ceftriaxone and benzylpenicillin were selected for sampling as these were among the most administered parenteral antibiotic medicines among hospitalized patients in all the three facilities. We sampled six batches of ceftriaxone and three batches of benzylpenicillin. For each sampled medicine batch, at least ten vials or ampoules were collected for quality analysis. Unlike parenteral medicines which are issued on a daily or weekly basis from the main pharmacy, oral medicines are issued in bulk and kept within the wards for longer duration. Thus, it was difficult to confirm the batch which was administered to patients during their time of hospital stay. Oral antibiotic medicines were therefore not sampled and all patients who were given oral medicines, or a combination of both oral and parenteral medicines were removed from the analysis.

### Data collection and analysis

Patient management files for eligible participants were reviewed by investigators who were guided by senior clinicians. The clinicians interpreted clinical assessments and the patient’s laboratory results. Prior to the study inception, these clinicians were trained on pharmacovigilance principles and oriented on the study protocol by the investigators. Demographics and clinical data such as age, sex, stated diagnosis and prescribed antibiotics were collected. Suspected ADRs were detected using a global trigger tool by the investigators. This tool was developed by the Institute for Healthcare Improvement (IHI) in the United States of America to help optimize the retrospective detection of adverse events using inpatient hospital records. It applies the use of certain triggers or clues such as switching or ordering of new medicines, abnormal laboratory results, and changes in patient prognosis (Griffin and Resar, 2009). Suspected ADRs were subjected to causality assessment using the Naranjo criteria (Murali et al., 2021). We further classified patients based on their outcomes as to whether they had recovered or were recovering from the illness (Aguilera et al., 2021). Recovered or recovering patients were those whose admission complaints were alleviated or improved during treatment respectively. Not recovered patients were those who did not show any improvement, or were referred to a higher-level facility or died after treatment was started. Furthermore, we calculated the Charlson Co-morbidity Index (CCI) based on the presenting co-morbidities for each patient using the CCI algorithm. The CCI algorithm applies weighted scores depending on patient characteristics such as age and seriousness of presenting co-morbidities such as HIV/AIDs, history of cardiovascular accident and cancer (Glasheen et al., 2024; Hall et al., 2019).

Sampled parenteral antibiotic medicines were subjected to pharmacopeial assay for absolute quantification of the API. We adapted pharmacopeial assay methods from the United States Pharmacopoeia (The United States Pharmacopeia, 2024; Disha, 2013). For both benzylpenicillin and ceftriaxone, we used a stainless-steel column (25 cm × 4.6 mm) packed with octadecyl silyl silica gel (5 µm) (Reprisals C18) for analysis using Agilent^®^ 1120 HPLC instrument. The mobile phase for benzylpenicillin assay consisted of 6.8% w/v potassium dihydrogen phosphate and methanol adjusted to pH3 using orthophosphoric acid and detection was done at wavelength of 220 nm. For ceftriaxone, the mobile phase consisted of 0.2M potassium dihydrogen phosphate buffer combined with acetonitrile, and detection was done at a wavelength of 254 nm. Our methods were internally validated by ensuring that linearity of the standard curve *R* ^2^ > 0.995, tailing factor NMT 2, and good precision with RSD NMT 2%.

### Data management and statistical analysis

Data was entered and cleaned in Microsoft Excel 2019 version and imported to STATA 14.1 for statistical analysis. Laboratory test results for antibiotic medicine quality were entered as categorical data. For descriptive analysis, numerical variables were presented as means, medians and interquartile range while frequencies and percentages were calculated for categorical variables.

### Ethics statement

The studies involving humans were approved by This study was approved by the Institution Review Board of Kamuzu University of Health Sciences-Malawi (College of Medicine Research and Ethics Committee (COMREC) under study number P.10/21/3447. The studies were conducted in accordance with the local legislation and institutional requirements. The participants provided their written informed consent to participate in this study. No potentially identifiable images or data are presented in this study.

## Results

### Patient’s demographic characteristics

Of the 224 patients included in the study, 60.71% (n = 136) were female and 39.29% (n = 88) were male. The median age for females was 43 years (IQR 35–63 years) and 43.5 years (IQR 30–63.5 years) among males. The most common diagnosis was sepsis (26.34%, n = 59), followed by pneumonia (20.09%, n = 45) and meningitis (7.59%, n = 17) (Table 1). More patients were given ceftriaxone (79.5%, n = 178), than benzylpenicillin (20.5%, n = 46). Overall, the median Charlson co-morbidity index (CCI) among the patients was 1 (IQR 0–3.5). By sex, the median CCI was 1 (IQR 0–4) among female and 0 (0–3) among male patients.

**TABLE 1:** Patient’s demographic characteristics and prescribed antibiotics.

Variable	Characteristics	Frequency	Percentage (N = 224)
Age	Median, IQR	43	30–60*
Sex	Female	136	60.71
	Male	88	39.29
Charlson Score	Median, IQR	1	0–3.5*
Facility	Facility 1	96	42.86
	Facility 2	71	31.7
	Facility 3	57	25.45
Diagnosis	Sepsis	59	26.34
	Pneumonia	45	20.09
	Meningitis	17	7.59
	Cellulitis	8	3.57
	Peptic ulcers	7	3.13
	Others	88	39.29
Antibiotics prescribed	Benzylpenicillin	46	20.5
	Ceftriaxone	178	79.5

*Numerical summaries presented as Median and interquartile range (IQR).

### Quality of administered antibiotics

We collected samples of antibiotic medicines given to the patients in the study. A total of nine batches of parenteral antibiotic medicines (that included six batches of ceftriaxone and three for benzylpenicillin) were sampled and subjected to test for content of active pharmaceutical ingredients (API). Among the antibiotics tested, we found one sample of out of specification benzylpenicillin. This sample was claimed to originate from China and contained only 61.8% of the declared amount of API. The other samples of benzylpenicillin were from China and India and contained 95% and 98% of the declared API respectively. Ceftriaxone samples from all the three facilities were stated to be manufactured in India. The determined API contents for ceftriaxone samples ranged from 102%–120% (Table 2).

**TABLE 2:** Antibiotic samples and test results for API content.

Sample ID	Medicine name	ATC CODE^a^	Strength	Dosage form	MEML category^b^	WHO AWaRe classification^c^	Stated country of origin	Assay % determination
Cef 101	Ceftriaxone	J01DD04	1000 mg	IV/IM powder	DVA	Watch	India	116.9
Cef 102	Ceftriaxone	J01DD04	1000 mg	IV/IM powder	DVA	Watch	India	119
Cef 103	Ceftriaxone	J01DD04	1000 mg	IV/IM powder	DVA	Watch	India	120.3
Cef 104	Ceftriaxone	J01DD04	1000 mg	IV/IM powder	DVA	Watch	India	103
Cef 105	Ceftriaxone	J01DD04	1000 mg	IV/IM powder	DVA	Watch	India	110
Cef 106	Ceftriaxone	J01DD04	1000 mg	IV/IM powder	DVA	Watch	India	102.5
Benzyl 101	Benzylpenicillin	J01CE01	5MU	IV/IM powder	HVA	Access	China	95.4
Benzyl 102	Benzylpenicillin	J01CE01	5MU	IV/IM powder	HVA	Access	China	61.8^d^
Benzyl 103	Benzylpenicillin	J01CE01	5MU	IV/IM powder	HVA	Access	India	98

^a^ ATC = anaomical, therapeutic, and chemical classification (https://www.whocc.no/atc_ddd_index/).

^b^ Malawi Essential Medicines Lists (EML) classifies medicines according to the level of healthcare facilities where the medicines are expected to be found, the importance of the condition treated and procurement system. H = found at health centre, district hospital and central hospital levels; D = found at district hospital and central hospital levels only; C = found at central hospital level only. By importance, V = vital medicines and E = essential medicines. By procurement system A = medicines required by a large number of patients as such to be routinely procured and stocked by CMST and B = medicines required for a limited number of patients and not routinely stocked by CMST.

^c^ WHO classifies antibiotic medicines according to the risk of inducing resistance. Access categories comprise of antibiotics which are narrow spectrum and routinely used with low risk of resistance. Watch medicines are broad spectrum antibiotics with a higher risk of inducing resistance. Reserve antibiotics are the last resort used in multi-drug-resistant infections.

^d^ Extreme deviation with low API content.

### Patient clinical outcomes

**FIGURE 1:**
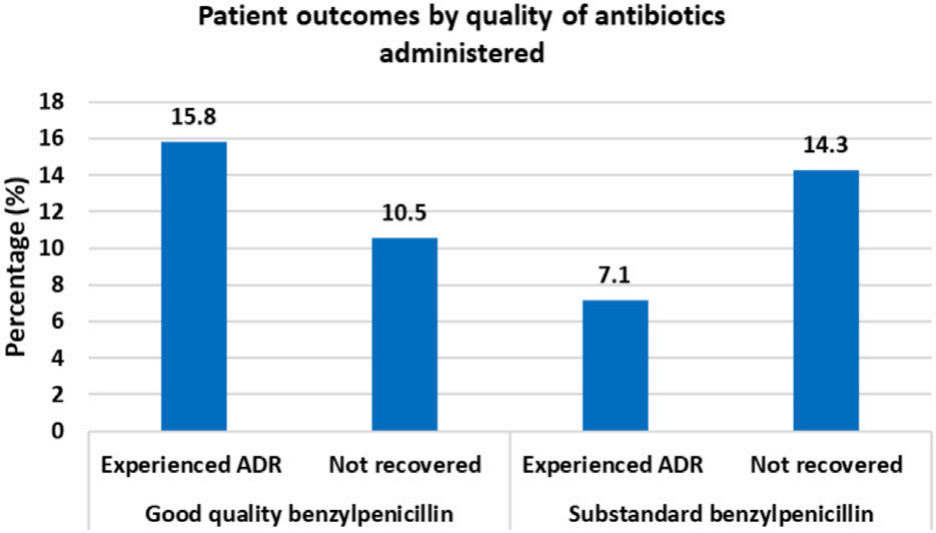
Clinical Outcomes for patients administered with benzylpenicillin of different quality status by the content of active pharmaceutical ingredients.

Of the 224 patients, suspected ADRs occurred in 18.3% (n = 41) of patients while 12.05% (n = 27) did not recover from their illness. By causality assessment, 2.4% (n = 1) were certain, 17.1% (n = 7) probable, and 80.1% (n = 33) possible ADRs (Naranjo et al., 1981). Among patients who received benzylpenicillin with optimal API content, 15.8% experienced ADRs while 10.5% failed to recover from illness. For patients who received benzylpenicillin containing lower than required amount of API, only 7.1% experienced an ADR while 14.3% failed to recover from illness (Figure 1).

Patient outcomes were significantly associated with age and CCI. The mean age among patients who did not recover was 55.7 years (95% CI 48.2–63.1 years) vs. 44.8 years (95% CI 41.9–47.7 years) among patients who recovered. Mean CCI was 3.1 (95% CI 2.0–4.2) among patients who did not recover vs. 1.8 (95% CI 1.5–2.2) among patients who recovered. Although there was a lower ADR rate among patients who were given antibiotics with a lower amount of API (7.1%) as compared to patients who were given antibiotics with optimal amount of API (18.5%), the difference was not statistically significant, p < 0.203 (Table 3). Similarly, the difference in failure to recover was not statistically significant between the group of patients who were given antibiotics with lower API content (14.3%) and who were given antibiotics with optimal amount of API (11.4%), p < 0.502.

**TABLE 3:** Clinical outcomes among patients and associated factors.

Association with occurrence of ADRs	Factor	Characteristic	With ADRs (N = 41)	Without ADRs (N = 183)
	Age	Mean ± SD	50.6 ± 24.2	45.1 ± 19.7
	Charlson score	Mean ± SD	1.92 ± 2.5	2 ± 2.2
	Antibiotic quality	Optimal API	34 (18.48)	150 (81.52)
		Low API	1 (7.14)	13 (92.86)
	Diagnosis	Sepsis	9 (15.25)	50 (84.75)
		Pneumonia	8 (17.78)	32 (82.22)
		Meningitis	6 (35.29)	11 (64.71)
		Cellulitis	2 (25)	6 (75)
		Peptic ulcers	1 (14.29)	6 (85.71)
		Others	15 (17.05)	73 (82.95)
	Antibiotics given	Benzylpenicillin	8 (17.39)	38 (82.61)
		Ceftriaxone	33 (18.54)	145 (81.46)

Association with patient recovery	Factor	Characteristic	Not recovered (N = 27)	Recovered (N = 197)
	Age	Mean ± SD	55.7 ± 18.7	44.8 ± 20.6
	Charlson Score	Mean ± SD	3.11 ± 2.7	1.83 ± 2.4
	Antibiotic quality	Optimal API	21 (11.41)	163 (88.59)
		Low API	2 (14.29)	12 (85.71)
	Diagnosis	Sepsis	5 (8.47)	54 (91.53
		Pneumonia	6 (13.33)	39 (86.67)
		Meningitis	1 (5.88)	16 (94.12)
		Cellulitis	1 (12.5)	7 (87.5)
		Peptic ulcers	2 (28.57)	5 (71.43)
		Others	12 (13.64)	76 (86.36)
	Antibiotics given	Benzylpenicillin	4 (8.7)	42 (91.3)
		Ceftriaxone	23 (12.92)	155 (87.08)

A multivariate logistic regression analysis was performed to assess the influence of possible confounders, but the results were not statistically significant.

## Discussion

In this study, we sampled and tested antibiotic medicines based on records of batches administered to our study patients. Medicine quality tests were conducted to quantify the API content using pharmacopeial HPLC methods for ceftriaxone and benzylpenicillin samples. By the API content, deviations from the specifications may be classified as either extreme or non-extreme; where extreme substandard medicines contain API of <80%, while non-extreme substandard medicines contain API between 80% to the lowest required content, which is usually (but not always) 90% (WHO; Hagen et al., 2020). Although all types of deviations in terms of pharmaceutical quality are undesirable, extreme deviations are more likely to cause harm such as adverse drug reactions, untreated diseases, prolonged hospitalization and death (WHO; Salami et al., 2023).

This study identified a batch of substandard benzylpenicillin with extremely low API content of 61.8% (United-States-Pharmacopeia-Convention, 2018; British-Pharmacoeia-Commission. British Pharmacopoeia, 2013). Benzylpenicillin is an important antibiotic and used for the treatment of serious infections in Malawi including neurosyphilis, meningitis and sepsis (Ministry-of-Health. Malawi Standard Treatment Guidelines, 2024). Thus, the availability of poor quality benzylpenicillin on the market poses a huge risk to the public health. A recent study conducted in Rumphi, Malawi also showed that penicillin antibiotics had the highest prevalence of resistance underscoring the need for efforts to safeguard the safety and efficacy of commonly used antibiotics such as benzylpenicillin in the country (Mithi et al., 2024). Currently, there is limited literature on the direct clinical impact of substandard antibiotic medicines on patient recovery and occurrence of adverse drug reactions. The current preliminary findings therefore highlights important signals on the potential impact substandard antibiotics especially in LMICs where the prevalences of both substandard antibiotics and antibiotic resistance are very high (Murray et al., 2022). Using data modelling techniques, it is estimated that about 169,000 pneumonia deaths per year are caused by poor quality antibiotics if the prevalence substandard antibiotics is at 10%. Thus, with the reported high prevalence of substandard antibiotics in Malawi, the estimated related death are mostly likely to be more than fold (World-Health-OrganizationWHO, 2017).

Most studies on the quality of antibiotics have assessed oral dosage forms such as amoxicillin, ciprofloxacin and metronidazole, with few studies have reporting on the quality of parenteral antibiotics such as ceftriaxone and benzylpenicillin (PATH, 2014). Generally, the prevalence of substandard oral antibiotics has been reported to be very high in LMICs including Malawi but a study in Rwanda, showed that parenteral antibiotics contributed to only 5.1% of the substandard antibiotic medicines (Asrade Mekonnen et al., 2024). This could be consistent with the findings in this study where we only detected one sample of substandard benzylpenicillin. However, this may also be attributable to the small sample size of tested batches in the current study. Furthermore, similar to other studies (Asrade Mekonnen et al., 2024), we only tested for API content which is the most principal parameter for parenteral antibiotics while oral antibiotics are usually also subjected to other tests such as the release of the API from the formulation. As such, even if the API content is optimal, other oral antibiotic samples fail based on disintegration or dissolution tests (Ozawa et al., 2018; Chiumia et al., 2022).

Augmented ADRs, which are common and related to the pharmacology of the API, pose a challenge to be linked to the quality of medicines as they present with signs and symptoms that are similar to other diseases. Similarly, lack of response to treatment (sometimes referred to as type F ADRs) may also be a challenge to be attributable to poor quality medicines because they are affected by several other possible factors (Shrestha et al., 2017; Sam et al., 2021). However, cluster analysis of ADRs and lack of responses that were commissioned to in the WHO vigibase^®^ has proved to be helpful in the detection of several cases of substandard medicines including quetiapine which was failing to release the API (dissolution) and salbutamol which was being sold after expiry date in the United States of America (Pozsgai et al., 2022; Juhlin et al., 2015).

In our study, we found a plausible pattern of occurrence of ADRs and patient recovery. Although the findings were not statistically significant, we observed lower rates of both ADR occurrence and patient recovery among patients who were administered with benzylpenicillin containing sub-optimal amount of API as compared to patients who were administered with benzylpenicillin with optimal API content. This requires confirmation in a bigger study as it suggests that sub-therapeutic doses of antibiotics may indeed potentially contribute to lower recovery rate (Appaneal et al., 2021; Shafrin et al., 2022), but less side effects which is due to the loss of efficacy. Ceftriaxone samples contained API content near to the upper limit (between 117% and 120%) and were also observed to have a higher prevalence of ADRs. There was, however, no further evidence that the observed ADRs were dose dependent.

Significant predictors of ADRs and recovery were age and CCI. Adverse drug reactions are common among elderly patients because the pharmacokinetics of most medicines are affected due to hepatic and renal dysfunction that is associated with aging (Muirhead et al., 2002; Helldén et al., 2013). The CCI is an indicator of disease complexity based on presence of important co-morbidities (Hall et al., 2019). It is therefore applied in studies to predict mortality among patient study participants (Charlson et al., 2022). The criteria consist of a series of questions for the presence of targeted and usually serious comorbidities such as HIV/AIDS, diabetes and cancers. The responses are weighted, and a final score is given based on the responses. Patients with a high CCI score are also more likely to take concomitant medicines which may potentially interact with antibiotic medicines and affect response and occurrence of adverse drug reactions (Arabyat et al., 2021; Brandariz-Núñez et al., 2023).

### Limitations

There was a sampling bias in this study as antibiotics were sampled based on exposure of the batch to the patients. Thus, a limited number of samples were collected and tested. The study also only focused on the API content, and did not test for other equally important quality parameters for parenteral medicines such as sterility and endotoxin levels. Further, the study also included a limited number of participants as this was also limited by the availability of records for tracing batches which were administered to patients. In the study, we did not adequately investigate other possible contributors to the lack of patient recovery and thus, the results may be biased and affected by other confounding factors. Furthermore, the use of retrospective and routinely collected data poses a challenge of missing out important information due to possible insufficient documentation and lack of verification or direct follow up with the patients. Due to the type of study design, there was a limited number of participants who were exposed to substandard antibiotics and therefore the study power was inadequate to accurately detect statistically significant effects. Thus, the findings of this study cannot be generalized to a larger population.

## Conclusion

We assessed the quality of parenteral antibiotic medicines sampled from three hospitals in southern Malawi and identified a batch of benzylpenicillin with extremely low API content. Patients who received benzylpenicillin with low API content presented with lower recovery rates and adverse events. Although this pattern is biologically plausible, the differences between were not statistically significant, underscoring the need for a larger prospective study with expanded quality parameters to further validate our findings. We recommend increased awareness and vigilance on the quality of antibiotic medicines as one of the key measures for mitigating antibiotic resistance.

## Data Availability

The original contributions presented in the study are included in the article/Supplementary Material, further inquiries can be directed to the corresponding author.
