# Supplementary material for: Substandard antibiotics and their clinical outcomes among hospitalized patients in southern Malawi: a pilot study
Source: Front Pharmacol. 2025 Mar 18;16:1535501. doi: 10.3389/fphar.2025.1535501 (PMC11959087; doi:10.3389/fphar.2025.1535501)
Supplement: Supplementary file 1 [file Image1.pdf]

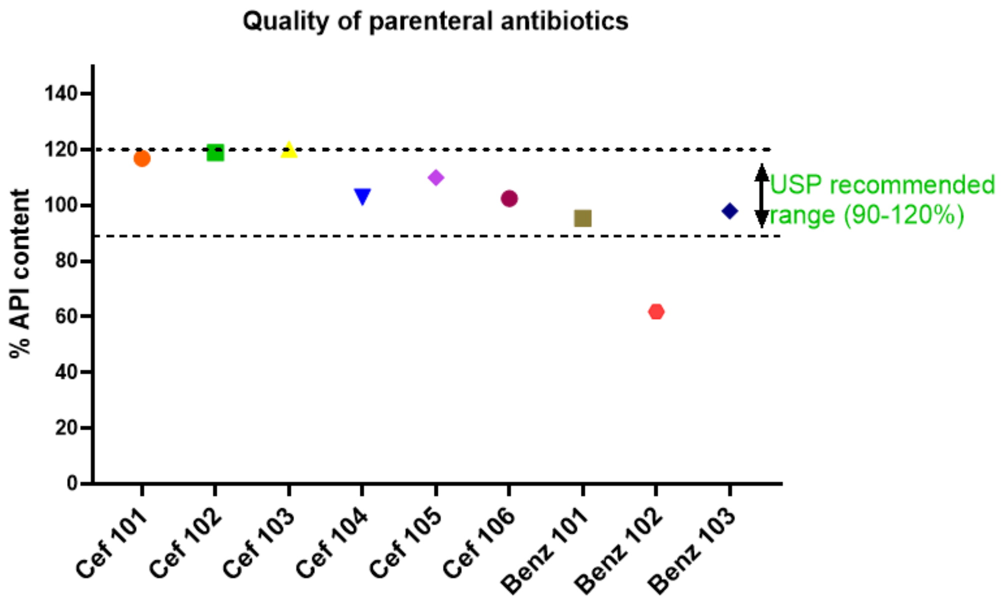

Supplementary figure 1. Assay results for medicine samples. The USP specifications for assay results for both ceftriaxone (Cef) and bezylpenicillin (Benz) is 90-120% of declared API content

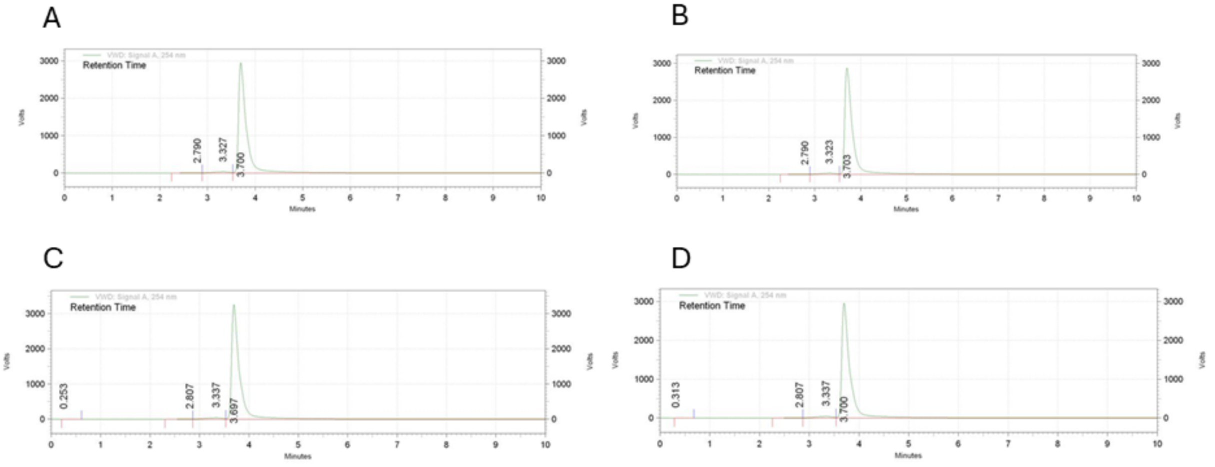

Supplementary figure 2. Representive chromatographic peaks for ceftriaxone standard (A), Sample code 104 from Zomba (B), Sample code 105 from Machinga (C) and sample code 106 from Nsanje (D)
